# Congenital epignathus associated with a cleft palate: a case report

**DOI:** 10.1186/s13256-021-03007-w

**Published:** 2021-08-03

**Authors:** Fabrice Lele Mutombo, Jason Nzanzu Kikuhe, Noé Kasereka Mwanamolo, Marc H. Erickson, Neil Wetzig, Médard Kabuyaya Kakule

**Affiliations:** 1 Surgery Department, Heal Africa Hospital, Goma, Democratic Republic of Congo; 2 ENT Department, GOMA Provincial Referral Hospital, Goma, Democratic Republic of Congo; 3 Surgery Department, Fort Memorial Hospital, Milwaukee, Wisconsin USA; 4 Faculty of Medicine, Surgery Department, University of Goma, Goma, Democratic Republic of Congo

**Keywords:** Epignathus, Teratoma, Cleft palate, Airway obstruction

## Abstract

**Background:**

Epignathus is a rare, benign, congenital teratoma of the hard palate with an estimated incidence of 1 in 200,000 live births. Epignathus frequently leads to a high mortality (80–100%) due to airway obstruction in the neonatal period.

**Case presentation:**

We report a case of successful management of a rare oropharyngeal teratoma in a African newborn girl who was referred to our institute with a large protruding intraoral mass, combined with cleft palate, causing some respiratory compromise. The palatal mass was removed on the fifth day after birth, and a palatoplasty performed on day 30.

**Conclusion:**

Epignathus is a life-threatening condition at the time of delivery. Appropriate management begins with securing the airway, followed by complete resection of the tumor.

## Introduction

Epignathus is a rare teratoma that arises from the oral cavity, most commonly from the palate. Congenital tumors of the oral cavity frequently affect normal development of adjacent structures, and tumors developing during morphogenesis of the palate are commonly associated with a cleft palate [1].

In general, teratomas are uncommon malformations that arise in the three embryonic germ layers (ectoderm, mesoderm, and endoderm). They are felt to be the result of pluripotent cells that did not complete migration from allantois to the genital ridge during the fourth and fifth weeks of gestation [2].

The physiologic impact of a teratoma is a result of its location and size. If located in the upper aerodigestive tract, they are high risk, frequently causing life-threatening complications from compromise of the airway. Because of this, they have a high mortality rate, and prenatal diagnosis may allow for better preparation to help optimize care at the time of birth [3–5].

Some teratomas extend beyond the mouth, and these lesions can be associated with mid-face defects including cleft lip and palate. There are reports of palatal teratomas extending beyond the oral and nasal cavity and even extending into the cranial cavity with resultant destruction of brain tissue [3, 6].

We have reported a case of congenital teratoma arising from the hard palate in a newborn girl. This report aims to show that diagnosis and successful surgical treatment of such a rare case are possible in a low-resource environment. It therefore contributes to the existing literature on management of giant epignathus.

## Case

**Fig. 1:**
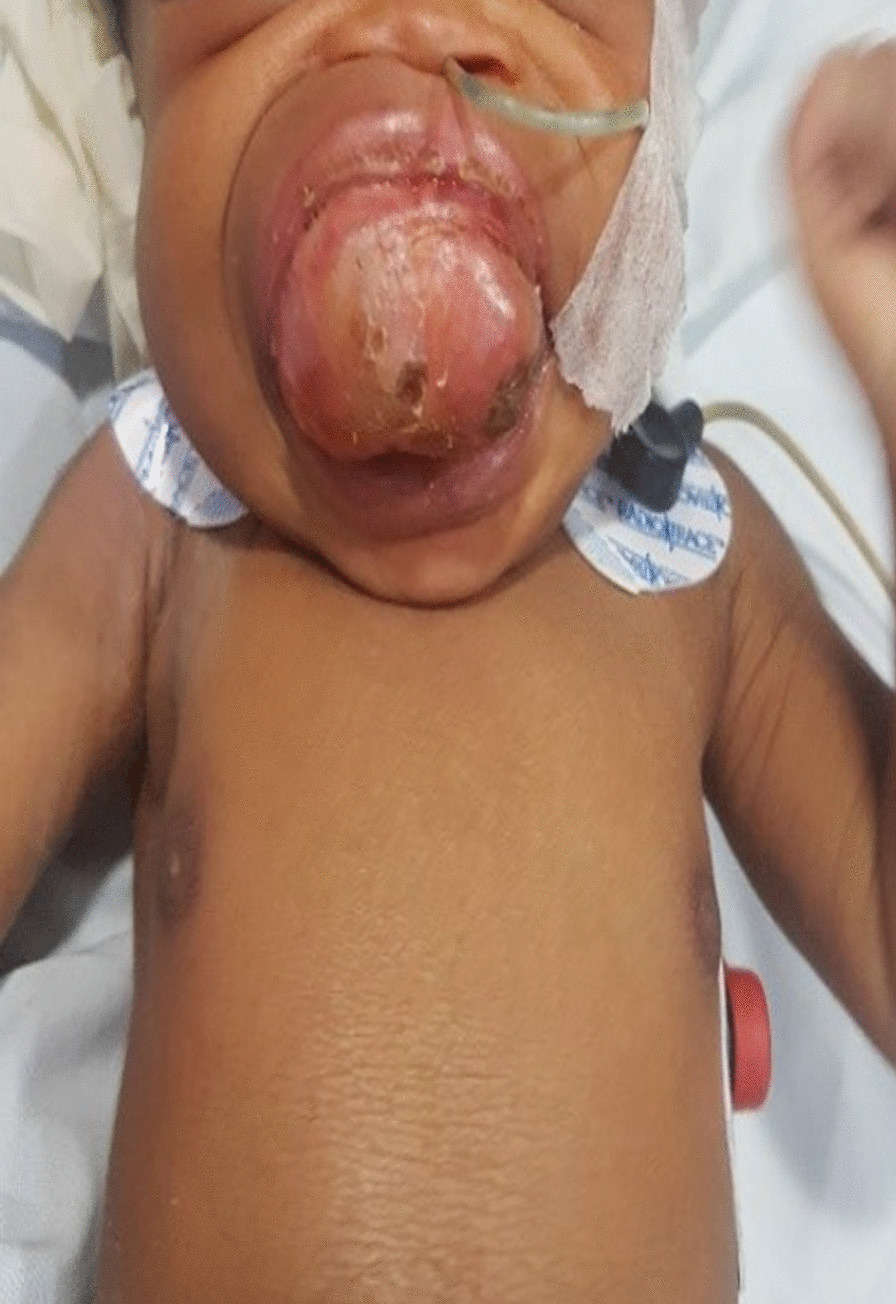
One-day-old female baby with an oropharyngeal mass

A 1-day-old female African newborn weighing 3500 g was referred for management of a large oral mass that protruded from the mouth and caused maxillary protrusion and flattening of the nose. The patient was having difficulty breathing (Fig. 1).

She was born by spontaneous vaginal delivery to a mother who was unaware of the date of her last menstrual period. She had no prenatal medical care, so there was no opportunity for prenatal diagnosis.

Initial examination revealed a mass that occupied the majority of the oral cavity and protruded from the mouth. On palpation, the mass appeared to arise from one side of the palate, and there was an adjacent palpable cleft. No other congenital anomalies were evident on physical examination.

The patient had some breathing difficulty, but the airway was patent. She was administered oxygen and intravenous fluids, and a nasogastric feeding tube was successfully placed. Feeding was commenced at 35 ml, eight times per day, and she stabilized.

On day 5, she was taken to surgery. Laryngoscopy was performed using a standard Macintosh blade size 1, and an easy intubation with a 2.5 endotracheal tube. The mass was excised under general anesthesia, Good hemostasis was achieved, and complete resection of the tumor was accomplished. There were no immediate postoperative complications.

**Fig. 2:**
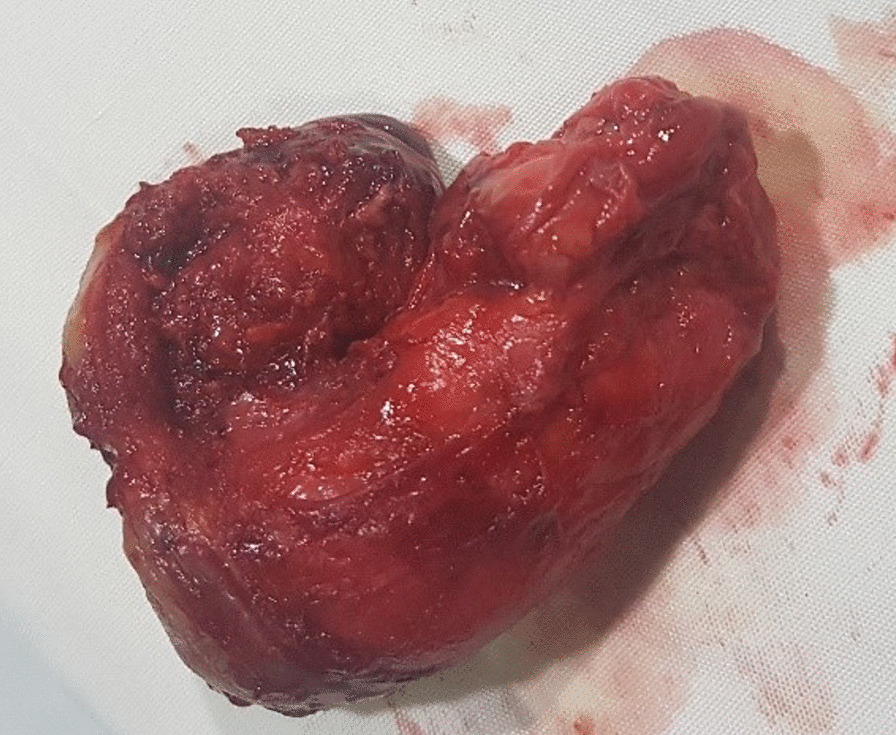
Tumor after total excision

Gross examination revealed a large fibrocystic tumor involving the oral and nasal cavities with its base originating from the hard palate. It measured 6 × 5 × 4.5 cm and was well circumscribed (Fig. 2).

Histopathologic examination revealed a congenital primarily epidermal teratoma.

**Fig. 3:**
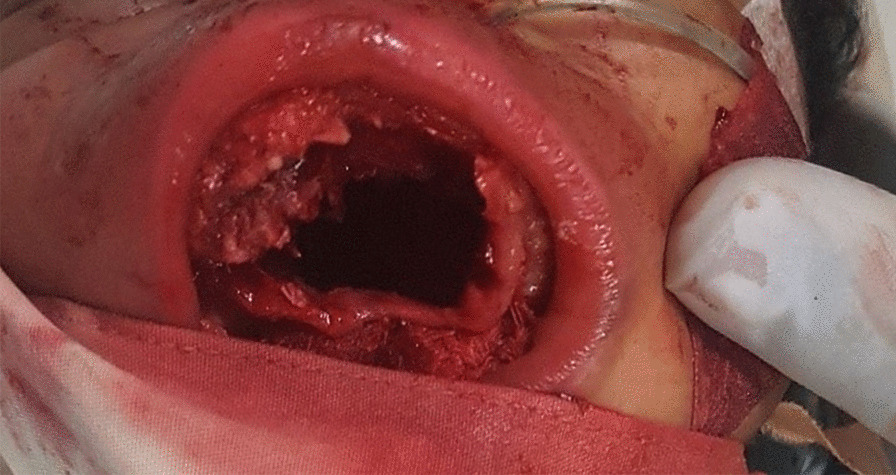
Five-day-old female baby, with a palatine defect post removal of palatine tumor

She had an uneventful postoperative course, although a palatal defect remained (Fig. 3). She had no difficulty opening or closing her mouth. Bottle feeding with expressed breast milk was initiated shortly after surgery and was well tolerated. She was able to breastfeed by postoperative day 5.

**Fig. 4:**
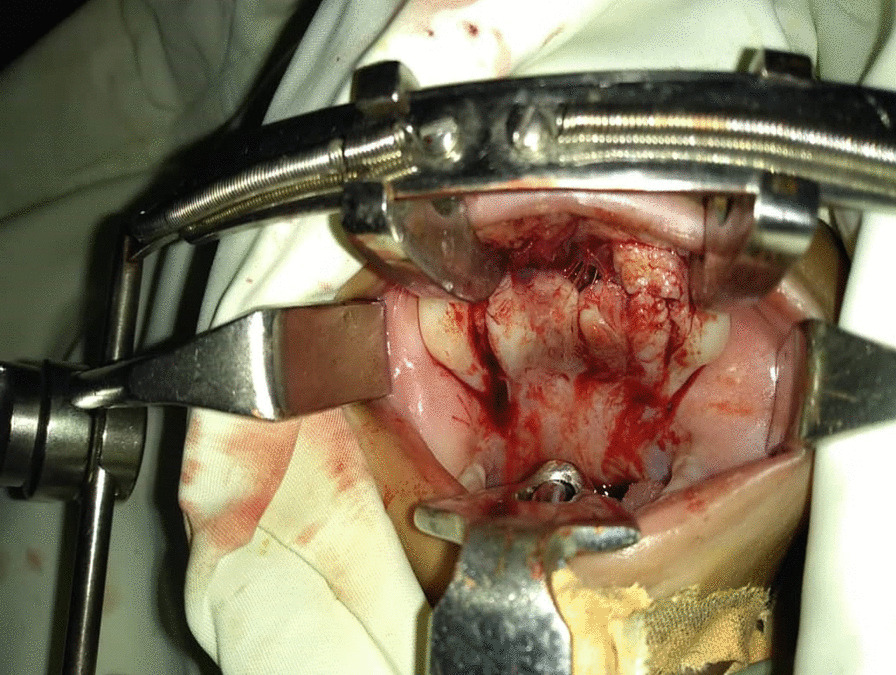
Reparation of palatine defect (intraoperative view)

**Fig. 5:**
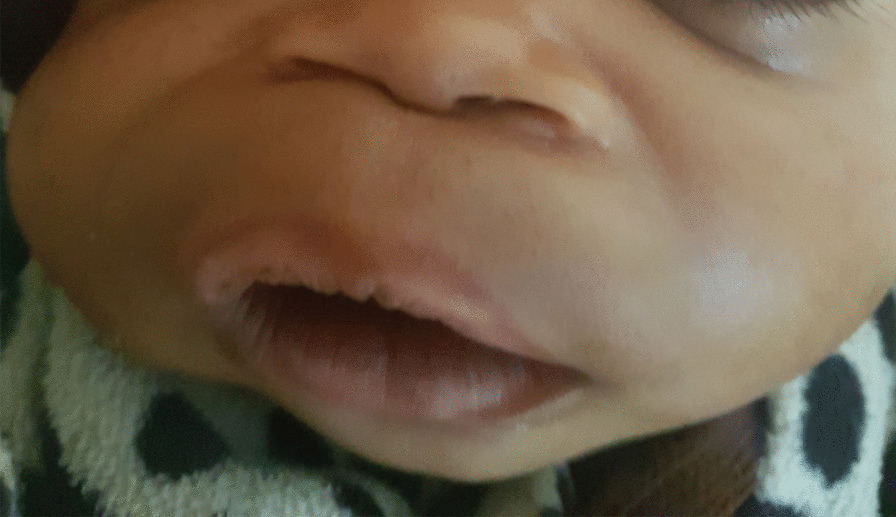
One-month-old female baby, with a palatine defect repaired

On postoperative day 30, the patient was taken back to surgery, where she underwent successful palatoplasty (Fig. 4). She was discharged home with her mother on postoperative day 9 after palatoplasty (Fig. 5).

## Discussion

Congenital oral tumors are commonly recognized at birth, but they can also be diagnosed antenatally by ultrasound or Magnetic resonance imaging (MRI). They can be solid or cystic and can be benign or malignant, but the majority are benign. *In utero*, obstruction of the oral cavity can result in polyhydramnios [7–9].

Teratomas occur in approximately 1 in 4000 births, and there is a female predilection. They are most commonly located in the sacrococcygeal area but can present in many areas of the body. Epignathus is very rare, occurring in only 1/200,000 live births. They account for only 2% of teratomas and almost always present in neonates. Most are benign, although malignancies have been reported [4–6]. Although other teratomas have a 6:1 female predilection, those of the oral cavity do not seem to have a clear sex predilection. In our case, the baby was female.

As reported by Kumar *et al.*, giant epignathus can lead to death during the neonatal period due to airway obstruction or can lead to malnutrition due to the inability of oral feeding. The newborn must therefore benefit from oxygenation and feeding by nasogastric tube while awaiting surgery. Complete surgical excision is the definitive treatment [5, 10].

Teratomas are often classified into four types:

1. *Dermoid*, also called “hairy polyps”, containing epidermal and mesodermal elements are the most frequent;
2. *Teratoid*, composed of ectodermal, mesodermal and endodermal elements, are very poorly differentiated;
3. *True teratoma*, also contain the three embryonic germ layers, but differentiated in recognizable structures such as cartilage, hair, teeth, etc.
4. *Epignathus*, are highly differentiated, rare, and have a high mortality rate due to their location [4].

Yoshimura *et al.* [11] proposed an alternative classification of teratomas with three types:

1. *Type 1* (skin and fatty tissue derived from two germ layers);
2. *Type 2* (teratoma with tissues representing the three germ layers, with bone, teeth, neural tissue, and tissues of the gastrointestinal tract); and
3. *Type 3* (parasitic twins with well-differentiated organs and limbs).

Histopathologic examination in this case revealed an epignathic teratoma containing mostly epidermal tissue. It was a type I teratoma by the Yoshimura classification system.

Oral teratomas can be associated with mechanical craniofacial abnormalities, the most common being cleft palate [12].

When a tumor develops before the eighth week, it can interfere with the normal closure of the secondary palate in the midline. It can also hinder the confluence of the two halves of the nose or tongue, resulting in a bifid nose or tongue. It can also contribute to abnormal maxillofacial development with micrognathia and ogival palate [13]. In our case, the baby had a nonfusion of the two palatal bones with resultant cleft palate.

## Conclusion

Epignathus is a rare oral cavity teratoma commonly presenting with symptoms requiring urgent management of respiratory distress.

As in this case, large epignathus can cause some respiratory compromise, and it is commonly associated with cleft palate, but other anomalies may occur.

Urgent treatment consists of airway management and nasogastric feeding tube. The definitive treatment requires complete resection of the mass and correction of associated anomalies.

## Data Availability

Not applicable.

## Abbreviations

IV: Intravenous
MRI: Magnetic resonance imaging
ENT: Ear, nose, and throat

## References

[1] Omezzine M, Bouslama S, Nouri S, Moatamri R, Khochtali H. Tumeurs congénitales de la cavité buccale et fente palatine: présentation de 2 cas. Med Buccale Chir Buccale. 2011;17(2):167–9. 10.1051/mbcb/2011109.

[2] Kundal V, Gajdhar M, Sharma C, Kundal R. Intraoral teratoma in a newborn presenting as severe respiratory distress. BMJ Case Rep. 2013;2013(251):2013008735–2013008735. 10.1136/bcr-2013-008735 PMC3618837 23531931

[3] Meziane M, Eabdenbitsen A, Boulaadas M, Essakalli L, Kzadri M. Double tératome de la cavité buccale? Med Buccale Chir Buccale. 2012;18(4):383–5. 10.1051/mbcb/2012039.

[4] Al-Mahdi A, Al-Khurrhi LE, Atto GZ, Dhaher A. Giant epignathus teratoma involving the palate, tongue, and floor of the mouth. J Craniofac Surg. 2013;24(1):e97–9. 10.1097/SCS.0b013e3182798f25 23348357

[5] Menezes Filho MP, Simao NMMS. Giant epignathus of the palate: a case report. J Bras Patol Med Lab. 2015. 10.5935/1676-2444.20150055.

[6] Kirishima M, Yamada S, Shinya M, Onishi S, Goto Y, Kitazono I, Hiraki T, Higashi M, Hida AI, Tanimoto A. An autopsy case of epignathus (immature teratoma of the soft palate) with intracranial extension but without brain invasion: case report and literature review. Diagn Pathol. 2018;13(1):99. 10.1186/s13000-018-0776-y PMC6303979 30579363

[7] Bonet C, Peñarrocha-Oltra D, Minguez JM, Vera-Sierra B, PeñarrochaDiago M, Peñarrocha-Diago M. Oral teratomas: a report of 5 cases. J Oral Maxillofac Surg. 2012;70:2809–13. 10.1016/j.joms.2012.01.028 22883319

[8] Fister P, Volavsek M, Novosel S, Jazbec J. A newborn baby with a tumor protruding from the mouth. Diagnosis: congenital gingival granular cell tumor. Acta Dermatovenerol Alp Panon Adriat. 2007;16:128–30. 17994174

[9] Benson R, Fabbroni G, Russel J. A large teratoma of the hard palate: a case report. Br J Oral Maxillofac Surg. 2009;47(1):46–9. 10.1016/j.bjoms.2007.12.015 18602201

[10] Kumar S, Shrilkrishna U, Shetty S, Sitaram A. Epignathus with fetiform features. J Lab Phys. 2011;1(3):56–8. 10.4103/0974-2727.78571 PMC3118061 21701667

[11] Yoshimura H, Maeda K, Yamamoto T, Itoh H. Epignathus: two case reports and a review of neonatal cases in Japan. Jpn J Pediatr Surg. 1988;20:607–12.

[12] Levine AB, Alvarez M, Wedgwood J, Berkowitz RL, Holzman I. Contemporary management of a potentially lethal fetal anomaly: a successful perinatal approach to epignathus. Obstet Gynecol. 1990;76:962–6. 2216267

[13] Benouaiche L, Couly G, Michel B, Devauchelle B. Diagnostic et prise en charge des tératomes cervico-faciaux congénitaux: à propos de quatre cas, revue de la littérature et mise au point. Ann Chir Plast Esth. 2007;52:114–23. 10.1016/j.anplas.2006.08.007 17030386

